# CARDIS (Cardiac ARrest DIgital Support): study protocol for a randomised controlled trial of a web-based support intervention for cardiac arrest survivors

**DOI:** 10.1016/j.resplu.2026.101235

**Published:** 2026-01-18

**Authors:** Annette Waldemar, Johan Israelsson, Katarina Heimburg, Erik Blennow Nordström, Per Nordberg, Anders Bremer, Kristofer Årestedt, Ingela Thylén

**Affiliations:** a Department of Health, Medicine, and Caring Sciences, Linköping University, Linköping, Sweden; b Clinical Department of Cardiology, Region Östergötland, Norrköping, Sweden; c Faculty of Health and Life Sciences, Linnaeus University, Växjö, Sweden; d Department of Internal Medicine, Kalmar County Hospital, Region Kalmar County, Kalmar, Sweden; e Department of Clinical Sciences Lund, Neurology, Lund University, Lund, Sweden; f Department of Cardiology, Skane University Hospital, Lund, Sweden; g Department of Rehabilitation Medicine, Skåne University Hospital, Sweden; h Department of Physiology and Pharmacology, Karolinska Institutet, Stockholm, Sweden; i Department of Clinical Science and Education, Centre for Resuscitation Science, Södersjukhuset, Karolinska Institutet, Stockholm, Sweden; j Function Perioperative Medicine and Intensive Care, Karolinska University Hospital, Stockholm, Sweden; k Department of Research, Region Kalmar County, Kalmar, Sweden; l Clinical Department of Cardiology, Region Östergötland, Linköping, Sweden

## Abstract

**Background:**

Surviving sudden cardiac arrest often lead to long-term cognitive, emotional, and physical consequences. Although clinical guidelines recommend structured post-cardiac arrest follow-up, such follow-up is often lacking or inconsistent. Tailored digital interventions are scarce but may help address gaps in follow-up resources. The CARDIS trial evaluates the effects on patient-reported outcome measures of a co-created, web-based support programme designed to improve wellbeing, self management, and reintegration into everyday life for cardiac arrest survivors.

**Methods:**

CARDIS is a multicentre, parallel-group, randomised controlled trial enrolling cardiac arrest survivors aged >18 years. Participants will be randomised 1:1 to intervention or control. Both groups will receive standard post-cardiac arrest care, including a routine followup visit with screening and management of cognitive, physical and emotional health, as well as a printed booklet. The intervention group will additionally receive access to a webbased selfguided support programme for 3 months. After study completion, control participants will be offered the programme.

**Outcomes:**

Primary outcome is overall wellbeing and health (QWB) at 3 months. Secondary outcomes include self-reported cognitive problems, HRQoL, life satisfaction, symptoms of depression and anxiety, post-traumatic stress, fatigue, and sleep disturbances. A process evaluation will evaluate social selection bias, adherence and participants experiences.

**Discussion:**

The CARDIS trial will investigate the use of more accessible and standardised follow-up pathways by complementing existing care structures, thereby enhancing equity in long-term recovery and quality-of-life without requiring additional healthcare resources. The process evaluation will provide data on adherence, social selection, and engagement, essential for future implementation.

**Trial registration:** The trial is registered at clinicaltrials.gov (NCT07240714).

## Highlights

• CARDIS tests a co-created web-based support programme for cardiac arrest survivors.
• CARDIS has a randomised controlled design with a waiting list control group.
• Tailored content addresses emotional, cognitive, and physical needs.
• Primary outcome is wellbeing and health at 3 months post-enrolment.
• Process evaluation explores adherence, social selection, and participant engagement.

## Introduction

Sudden cardiac arrest is a leading cause of premature death worldwide.^1^ Each year, approximately 300,000 out-of-hospital cardiac arrests (OHCA) occur in Europe, with survival averaging 7.5 %. In contrast, in-hospital cardiac arrest (IHCA) affects 1.5–2.8 per 1000 hospital admissions, with 30-day survival ranging between 27 % and 62 %.^2^

Most cardiac arrest survivors reported in clinical trials and registries are considered to have a favourable neurological outcome.^3^, ^4^, ^5^ However, survivors often face long-term cognitive, physical, psychological, and social sequelae, including impaired daily functioning and reduced health-related quality of life (HRQoL) that may persist for months or years.^6^, ^7^, ^8^, ^9^, ^10^, ^11^, ^12^ Many survivors of working age remain on long-term sick leave due to residual consequences.^13^ Psychological problems such as anxiety, depression, sleep difficulties, and post-traumatic stress disorder (PTSD) are common, affecting up to one in four survivors.^14^, ^15^, ^16^ Cognitive impairment and fatigue are also frequent,^5^, ^17^, ^18^, ^19^, ^20^, ^21^, ^22^ further limiting the ability to resume previous activities and work.^23^, ^24^ These consequences place a substantial burden on individuals, families, and society. Yet, survivors frequently encounter fragmented follow-up, insufficient rehabilitation, and difficulty accessing reliable information,^25^, ^26^, ^27^, ^28^, ^29^ with family members experiencing emotional distress as they take on caregiving responsibilities.^23^, ^30^ Education about recovery has been ranked as the most important intervention to reduce caregiver burden.^31^ Collectively, these complex problems highlight the need for holistic care models extending beyond initial survivorship to long-term rehabilitation and support. Structured rehabilitation has been shown to improve HRQoL and facilitate return to work.^13^, ^32^, ^33^, ^34^, ^35^, ^36^

Existing post-resuscitation care guidelines recommend follow-up within three months of hospital discharge to assess physical, emotional, and cognitive health.^37^, ^38^ However, studies in Sweden have shown that follow-up is inconsistent, and especially patients with non-cardiac causes of cardiac arrest, or IHCA survivors, often receive none at all.^39^ Similar shortcomings are evident internationally, where follow-up is frequently restricted to medical issues, while psychological, cognitive, and social needs remain unmet.^13^, ^27^, ^28^, ^40^, ^41^ As a result, limited understanding of cardiac arrest and its consequences can contribute to uncertainty and distress,^42^ and feeling unprepared for discharge may increase the risk of readmission among cardiac patients more broadly.^43^ Digital solutions could offer a complementary and cost-effective means of support and education, enhancing accessibility regardless of underlying cause of cardiac arrest, access to rehabilitation services, or place of residence.

Despite growing interest in digital health, web-based interventions tailored specifically for cardiac arrest survivors remain scarce. Current initiatives include ENFORCER in Italy, evaluating a structured one-year rehabilitation programme,^44^ STEPCARE in the UK and Sweden, testing a clinician-led psychoeducational intervention for cardiac arrest survivors and their key family members,^45^ and the dyad feasibility studies Recovering Together after Cardiac Arrest (RT-CA) in the US^46^ and CARESS in the UK.^47^ However, all these programmes rely on guided clinician-led on-line sessions or structured schedules. There remains a need for flexible digital solutions that accommodate individual recovery trajectories and diverse care contexts, enabling patients to engage at their own pace and according to individual needs.

**CARDIS** (**C**ardiac **AR**rest survivors **DI**gital **S**upport) is designed to address this gap. The multicentre randomised controlled trial (RCT) evaluates a co-created, user-friendly, self-guided web-based platform in Swedish, enabling survivors of both IHCA and OHCA – with or without a cardiac cause – to access tailored content, set personalised recovery goals, and engage with evidence-based health information and self-management strategies addressing emotional, cognitive, and physical consequences of cardiac arrest.

## Methods

### Co-creation of the intervention

**Fig. 1:**
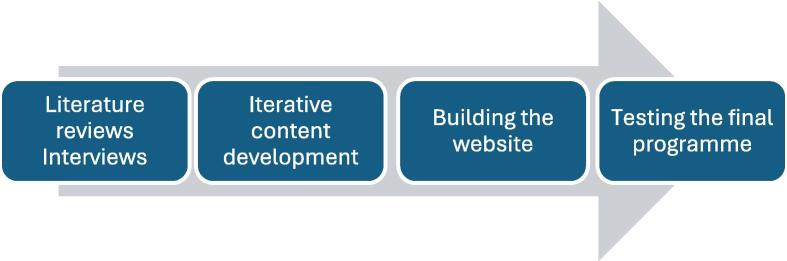
**Co-creation of the intervention**.

The web-based support programme was co-created in 2024–2025, using an iterative and user-centred design approach,^48^ in collaboration with the Swedish HeartLung Association and the Swedish Network for Cardiac Arrest Survivors and their Relatives. This approach ensured that the needs and lived experiences of cardiac arrest survivors guided the development process. An overview of the co-creation process is illustrated in Fig. 1.

#### Needs assessment through interviews

Focus groups and individual interviews involving 20 participants (November 2024 to January 2025) explored survivors’ experiences, support needs, and preferences for digital solutions to complement existing follow-up care. Participants highlighted the importance of varied formats (images, short and easy-to-read texts, hyperlinks, videos), accessibility across devices (smartphones, tablets, and computers), and subtitled/multilingual content. Suggested key topics included facts about cardiac arrest, hospitalisation, life after cardiac arrest, common symptoms and limitations, existential concerns, treatment, lifestyle, intimacy and relationships, sleep, coping, return to work, and peer support. Participants also emphasised the need for dedicated content tailored to co-survivors and bereaved.

#### Content development

The research team, survivors and family members collaborated with experts in communication (web designer, photographer, and communicator) and cardiac arrest care (nurses and physicians in cardiology, intensive care and rehabilitation, physiotherapist, occupational therapist, rehabilitation coordinator, psychologist, dietician, and chaplain) to co-create the content and structure of the programme. Draughts of texts and videos were reviewed by experts and end users. Following this, individual interviews were conducted with five survivors and three family members to gather feedback on the clarity, relevance, and tone of the texts and videos, including language and phrasing. The material was subsequently revised in accordance with their suggestions to ensure that the content was perceived as meaningful, accessible, and appropriate for the target audience.

#### Final programme structure and technical implementation

**Fig. 2:**
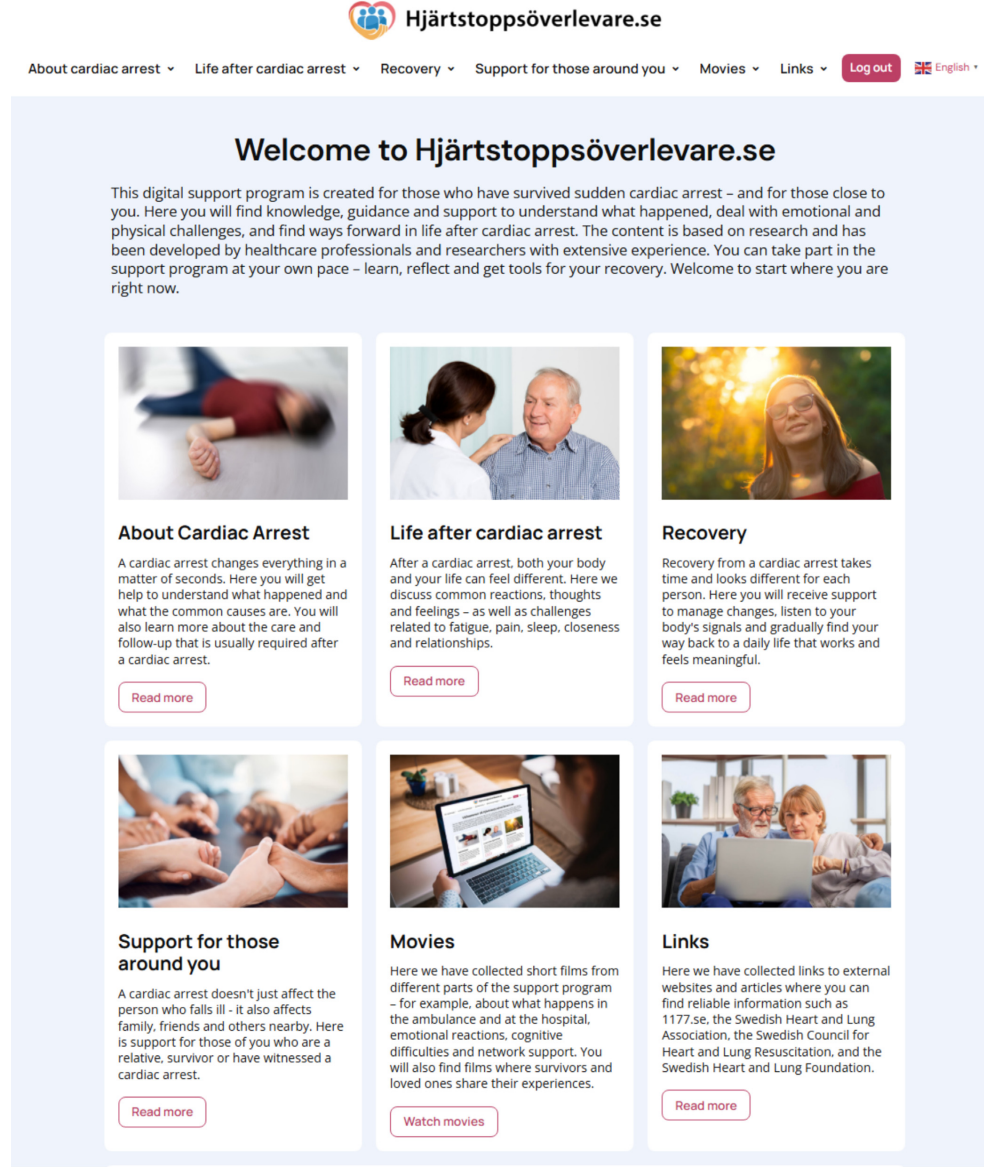
**Screenshot from the web-based support programme at****www.hjartstoppsoverlevare.se****, automatically translated from Swedish to English using GTranslate**.

The final version of the web-based support programme consists of four modules comprising 40 interrelated sections, addressing cardiac arrest and post-cardiac arrest care, physical recovery, psychological wellbeing, cognitive challenges, fatigue, intimate relationships, health behaviours and cardiovascular risk, and reintegration into daily life. One module targets co-survivors and children, also including bystanders and bereaved. Each module follows a consistent structure and includes written content, patient and family member narratives, short videos, images, animations, and links to validated external websites. Practical exercises are also suggested throughout the programme, such as mindfulness, breathing techniques, and strategies to reduce fatigue and promote physical activity. Additional features include support for creating an individual health plan and access to a moderated peer support chat forum (Fig. 2).

Readability was enhanced using the Lix programme (https://www.lix.se/). Particular attention was paid to ensuring that texts, images, and videos reflect the diversity of survivors' life situations and personal needs. The primary language of the programme is Swedish. To enhance accessibility for non-Swedish speaking users, an automatic translation plugin (GTranslate) was integrated into the website, enabling multilingual functionality across >100 languages, covering over 99 % of global internet users.^49^ Only text-based content within the programme is automatically translated; embedded videos, patient narratives, and external links remain in their original language. The programme was developed in WordPress, hosted at https://www.hjartstoppsoverlevare.se (login required during study), and will later be publicly available via the national patient organisation website (https://www.hjart-lung.se/).

#### Feasibility study

A feasibility study (*n* = 6; median age 60; median time since cardiac arrest 42 months) was conducted in August 2025 to assess the usability, acceptability, and technical functionality of the web-based support programme. Using cognitive interviews and the think-aloud method,^50^ participants navigated the programme while verbalising their experiences, with a researcher present to prompt reflection.

Participants described the content as relevant, clear, and emotionally supportive. The System Usability Scale (SUS)^51^ yielded a mean score of 87/100, indicating a very high perceived usability. Users engaged with the programme sequentially, selecting content based on personal relevance. The programme was consistently described as trustworthy, relatable, and more engaging than conventional written materials. The module for co-survivors was particularly valued, enhancing understanding of their experiences during and after ICU care. Also, content on cognitive impairment, fatigue, PTSD, and anxiety was especially appreciated. Participants praised the intuitive design, readability, and calming visual elements. Participants with cognitive challenges found the layout accessible. Embedded videos supported users with mental fatigue and external links were perceived as credible. The option to translate the programme into multiple languages reinforced its inclusivity. Working-age participants highlighted the value of content on returning to work and navigating financial and bureaucratic systems. All participants stated they would recommend the programme to others, including healthcare professionals.

### Protocol

CARDIS is an investigator-initiated, clinically driven trial with no involvement or sponsorship from commercial entities. The study protocol was drafted according to SPIRIT guidelines.^52^, ^53^ Ethical approval was obtained from the Swedish Ethical Review Authority (ID: 2025-06113-01) and the study is registered at https://www.ClinicalTrials.gov (NCT07240714).

#### Overall aim

The overall aim of this study is to evaluate the effectiveness of a web-based support intervention for cardiac arrest survivors, focusing on self-reported wellbeing, health, and everyday functioning. In addition, a process evaluation will explore social selection bias, adherence, and participant experiences. Research questions are listed in Table 1.

**Table 1:** Research questions related to the effect and process evaluation of the digital support programme.

**Research questions related to the effect evaluation**
1. What impact does the intervention have on overall wellbeing and health?
2. How does participation affect self-reported HRQoL, cognition, life satisfaction, symptoms of anxiety and depression, PTSD, fatigue, and sleep disturbances?
3. To what extent does self-rated health literacy moderate the effect of the intervention?
4. What influence does the intervention have on general self-efficacy?
5. How does engagement with the intervention and self-management relate to self-reported healthcare utilisation and return to work or studies among individuals of working age?

**Research questions related to the process evaluation**

1. What level of adherence do participants demonstrate during the 3-months intervention period?
2. How adherent are participants in completing outcome measures at 3- and 6-months?
3. Are there differences in background characteristics between individuals who accept versus decline participation, and between those who complete versus discontinue the study?
4. How does self-rated health literacy correlate with adherence to the intervention?
5. How many eligible patients decline participation, and how many are excluded due to inclusion or exclusion criteria?
6. In what ways and how many minutes have participants in the intervention group engaged with the programme?
7. What experiences and perceived benefits are reported by participants who assessed the intervention?

### Study design and setting

CARDIS is a single-blinded (investigator blinded) parallel-group multicentre RCT. Participants will be recruited from eight sites with established post-cardiac arrest follow-up pathways. All participating sites will receive training to ensure that follow-up procedures align with standard care, as defined by current clinical guidelines.^37^, ^38^

Standard care includes screening and management of fatigue, cognitive function, and emotional and physical status, as well as providing information and support for survivors and their family members. As part of standard care, a printed informational booklet published by the Swedish Resuscitation Council^54^ is also provided. The booklet contains information on cardiac arrest, recovery, emotional and cognitive challenges, health-promoting behaviours, and available support resources.

The participating sites are located in regions with a combined population of approximately 6 million inhabitants, ensuring geographic and organisational diversity while supporting the feasibility of timely recruitment.

Based on historical data from the participating centres, the annual patient flow is expected to be approximately 350 patients, of which an estimated 30 % will be excluded due to eligibility criteria. Additionally, 30 % are expected to decline participation, leaving approximately 170 patients eligible for inclusion each year. The full sample size is therefore expected to be reached within 18 months of study initiation. Should recruitment proceed more slowly than anticipated, an extension of the enrolment period to 24 months will be requested.

### Eligibility criteria

Inclusion: ≥18 years of age, survived cardiac arrest 1–3 months prior to enrolment, access to internet-enabled device.

Exclusion: insufficient linguistic or cognitive ability to engage with the intervention or complete questionnaires designed in Swedish, or concurrent participation in another study collecting patient-reported outcome measures (PROMs) as primary endpoints.

### Study procedures

**Fig. 3:**
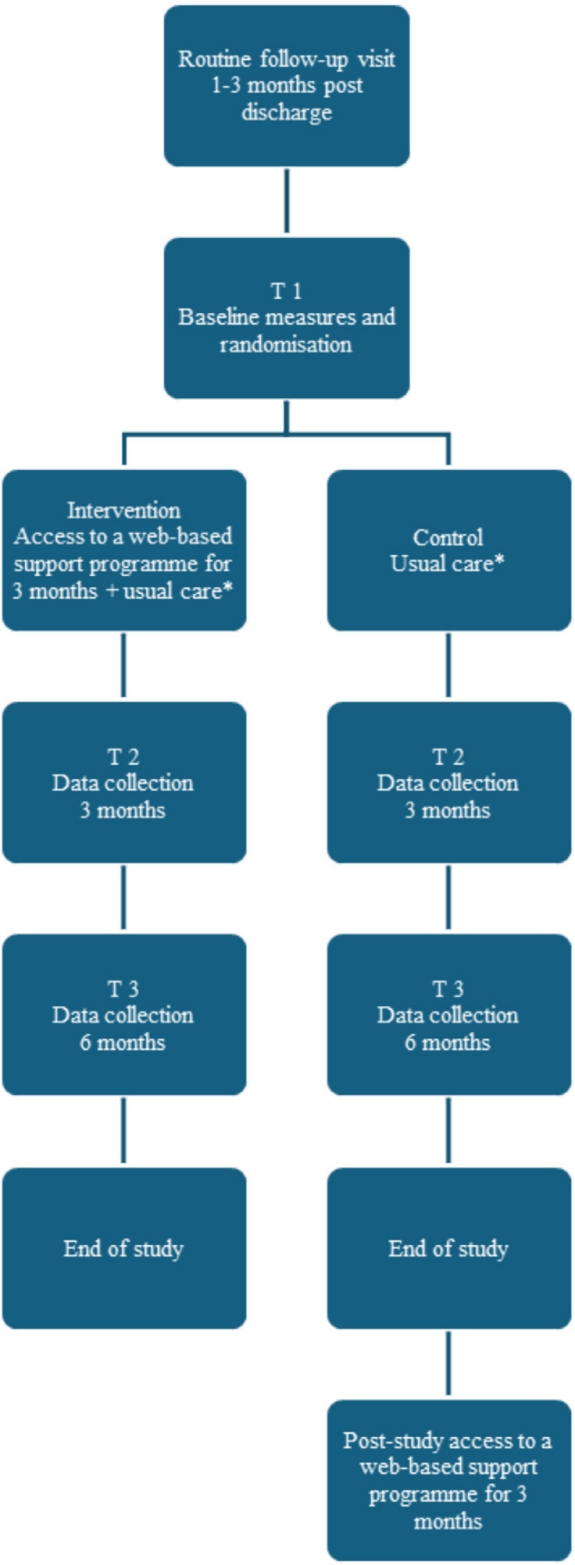
**Flowchart study procedure**. *Usual care = a printed informational booklet, one-time individual self-management advice, and standard post-cardiac arrest care as defined by current clinical guidelines.

An overview of the study flow is illustrated in Fig. 3.

### Recruitment and consent

Eligible patients will be identified by local healthcare professionals during routine follow-up 1–3 months post-discharge. Those meeting the eligibility criteria will receive verbal and written information about the study. Digital informed consent will be obtained by the national coordinator prior to data collection. Participants will be informed that participation is voluntary and that they may withdraw at any time without consequences for their care.

#### Randomisation

Participants will be randomised 1:1 to intervention or control using block randomisation stratified by site, ensuring balanced allocation within each site. Randomisation will be managed through REDCap (Research Electronic Data Capture), hosted at Linköping University. REDCap is a secure, web-based platform widely used in academic research for randomisation and electronic data capture.^55^, ^56^

The CARDIS trial is single-blinded. The national coordinator responsible for randomisation and survey distribution via REDCap is not blinded while data analysts and primary investigator will remain blinded to group allocation during data analysis. Allocation codes will be used in the exported dataset, and group identities will only be revealed after completion of all primary analyses. Participants and care providers are not blinded due to the nature of the intervention.

### Intervention group

Participants allocated to the intervention group will receive access to the web-based support programme (https://www.hjartstoppsoverlevare.se) in addition to receiving a printed informational booklet, one-time individual self-management advice and standard post-cardiac arrest care, as defined by current clinical guidelines.^37^, ^38^ The programme is self-guided and designed to be used flexibly, allowing participants to engage with the content according to their individual needs and pace over a 3-months period. Participants are permitted, but not required, to involve a key family member when taking part of the programme.

To promote adherence, participants allocated to the intervention group will receive automated reminders every third week encouraging continued engagement with the digital support programme throughout the access period.

The intervention is grounded in the concept of self-care maintenance, which refers to behaviours aimed at promoting well-being, preserving health, and maintaining physical and emotional stability.^57^ During the intervention, participants are encouraged to set personal goals and create a health plan for their rehabilitation aligned with the programme’s focus areas. Key content is listed in Table 2.

**Table 2:** Key content in the support programme promoting self-care maintenance.

*Cognitive support*	Information material on common cognitive sequelae (e.g., attention, memory, executive function) and strategies for managing them.
*Emotional support*	Guidance on emotional challenges such as anxiety, depression, post-traumatic stress disorder, fatigue, and sleep disturbances, with suggested exercises in relaxation, breathing, mindfulness, and socialisation.
*Physical rehabilitation*	Education on physical activity and its role in recovery, including practical guidance delivered via video and lectures. The module also highlights the risk of kinesiophobia – fear of movement or physical exertion – which may hinder rehabilitation.
*Lifestyle support*	Guidance on nutrition, alcohol, and nicotine use, with exercises in smoking cessation, alcohol reflection, and healthy eating habits.
*Relationships*	Encouragement to re-establish intimate and social connexions, including exercises to reflect on relationship changes, rebuild social confidence, and explore intimacy with partners.
*Social support*	Encouragement to engage with patient organisations and peer networks, including access to a moderated chat forum via the Swedish network for cardiac arrest survivors and their relatives.
*Personal health planning*	Structured guidance to help participants to reflect on their current situation, identify personal needs and strengths, and formulate meaningful recovery goals. The module is designed to promote autonomy and self-efficacy, and can be completed individually or in collaboration with a family member or healthcare professional.

### Control group

Participants in the control group will receive a printed informational booklet, one-time individual self-management advice, and standard post-cardiac arrest care, as defined by current clinical guidelines.^37^, ^38^ After study completion, participants in the control group will be offered full access to the web-based support programme.

### Harms

Although the intervention is low-risk, participants will be advised to contact the study team if they experience distress or technical issues. Any adverse events or protocol deviations will be recorded and reviewed by the research management team.

### Outcomes and assessments

The primary outcome is overall wellbeing and health between the intervention and control groups 3 months after enrolment.

Secondary outcomes are self-reported HRQoL, life satisfaction, symptoms of depression and anxiety, PTSD, fatigue, cognitive impairment, sleep disturbances, and self-efficacy between the intervention and control groups 3- and 6-months after enrolment.

Assessments will be conducted at baseline, 3 months, and 6 months using self-reported validated digital questionnaires and study specific questions. Data completeness will be automatically monitored, and participants will receive regular reminders to ensure timely data entry. Variables are summarised in Table 3, Table 4.

**Table 3:** Data collection and assessments.

**Variables**	**Outcome measures**	**Baseline**	**3 months**	**6 months**
***Background variables***				
Sociodemographic variables	Study specific questions	x		
Health literacy	Scale for Communicative and Critical Health Literacy (SCCHL)^60^	x		

***Primary outcome***				
Overall wellbeing and health	Questionnaire on Well-Being (QWB)^58^	x	x	x

***Secondary outcomes***				
Health related quality of life	EuroQol-5D-5L, Visual Analogue Scale^61^	x	x	x
Life Satisfaction	Life Satisfaction Questionnaire (LiSat-11)^62^	x	x	x
Symptoms of anxiety	Generalised Anxiety Disorder Scale (GAD-7)^63^	x	x	x
Symptoms of depression	Patient Health Questionnaire-9 (PHQ-9)^64^	x	x	x
Posttraumatic stress	Impact of Event Scale–Revised (IES-R)^65^	x	x	x
Fatigue	Modified Fatigue Impact Scale (MFIS-21)^66^	x	x	x
Cognition	Cognition (single item question, SIQ)^67^	x	x	x
Sleep	The Minimal Insomnia Symptom Scale (MISS)^68^	x	x	x
Self-Efficacy	General Self-Efficacy Scale (S-GSE)^69^	x	x	x
Healthcare utilisation	Study specific questions		x	x
Return to everyday life	Study specific questions		x	x

**Table 4:** Sociodemographic variables and study specific questions.

**Variables**	**Measures**	**Baseline**	**3 months**	**6 months**
Sociodemographic	sex, age, origin, residence, occupation, education	x		
Medical history	co-morbidities	x		
Cardiac arrest event	date, location, aetiology	x		
Clinical management care	intensive care, treatment, interventions	x		
Healthcare utilisation	follow-up, acute visits, hospitalisation, encounters, reasons, triggers		x	x
Return to everyday life	return to work/studies, physical activity, fear of recurrence, support		x	x

#### Process evaluation

Specific attention will be given to evaluate adherence and potential social selection bias.

*Social selectivity* will be collected by keeping logbooks in the screening process in patients who accept vs. decline study participation (age and gender) and calculate the number of patients who do not meet the inclusion criteria for the intervention.

*Adherence to the study* will be monitored by calculating the number of participants who complete the study, discontinue participation, or fail to complete all questionnaires.

*Adherence to the intervention* (number of accesses and total time spent on the digital programme in the intervention group) will be analysed using continuous log-data integrated into the platform.

*Experience of the intervention* will be analysed using study specific questions. These address frequency and mode of programme use (individual vs. together with a key family member), the specific components accessed, and perceived helpfulness for recovery and future outlook.

### Sample size calculation and statistical analysis plan

The trial is planned to include 120 participants. A sample size calculation was conducted for the primary outcome variable (QWB). An a priori sample size calculation was conducted using G*Power for a repeated measures design with two groups and three time points (baseline, 3 months, and 6 months). Assuming a medium effect size (*f* = 0.25), a significance level of 5 % (*α* = 0.05), and a statistical power of 80 % (power = 0.80, corresponding to *β* = 0.20), the analysis showed that a total of 86 participants (43 per group) is sufficient to detect statistically significant differences between groups over time. To account for an estimated attrition rate of 30 %, a total of 120 participants (60 per group) will be recruited.

Baseline characteristics will be compared using chi-square, *t*-tests, or appropriate non-parametric methods. The intervention effect will be evaluated using linear mixed models (LMM) including fixed effects for time, group, and their interaction to assess differential changes over time between groups. Both random intercepts and slopes will be considered to account for individual variability in baseline levels and change over time.

All analyses will follow the intention-to-treat principle, whereby participants are analysed in their originally assigned groups regardless of adherence to the intervention.

There is no formally established minimal clinically important difference (MCID) for the QWB scale. However, previous research has shown that a total score below 50 is associated with clinically significant psychological distress.^58^ Effect sizes will be evaluated using marginal *R*^2^ and conditional *R*^2^.

Secondary outcomes will be analysed exploratorily. Although no formal power calculation has been conducted for these outcomes, their inclusion aims to provide a broader understanding of the potential effects of the intervention.

Adjustments for multiple comparisons, such as Bonferroni corrections, will be considered to control the risk of type I error.

No interim analyses are planned, as there are no anticipated safety concerns and the intervention does not involve clinical risk.

### Data management and confidentiality

Pseudonymised data will be stored in REDCap at Linköping University. Access is restricted to authorised personnel via two-factor authentication, and all data are backed up at least weekly. Each participant is assigned a unique study ID, with code keys stored separately from source data. Quality control will be performed through random cheques by external monitoring. Usage statistics from the digital support programme (e.g., logins, module visits, time spent) will be automatically collected without personal identifiers and linked to the study ID. Statistical analyses will be conducted on fully anonymised data. Data will be handled in accordance with applicable data protection regulations and retained for 10 years after publication.

### Patient and public involvement and engagement

The development of the intervention and the formulation of the research question were informed by a co-creation process involving cardiac arrest survivors and co-survivors. End-users contributed to identifying priority needs, shaping the content and structure of the web-based programme, and refining the focus of the study. The selection of outcomes and instruments available in Swedish was guided by current clinical guidelines, the COSCA recommendations,^59^ and the research team’s expertise, while ensuring alignment with domains highlighted as important by survivors and co-survivors.

### Dissemination policy

The study results will be published in peer-reviewed journals and presented at national and international conferences. Findings will be shared with participating hospitals through workplace seminars and with patient organisations to support dialogue on clinical and patient-centred implications. Public awareness will be promoted via local media and digital channels (https://cardis.se/ and https://se.linkedin.com). Results will also be communicated to expert bodies such as the Swedish Resuscitation Council to inform future guidelines. In addition, dissemination will be supported by the Swedish HeartLung Association and the Swedish Network for Cardiac Arrest Survivors and their Relatives to ensure broad public access and patient-centred communication of the findings.

### Trial status

The trial is currently in the pilot phase to evaluate recruitment, enrolment, randomisation, intervention delivery and data collection. The first patient was enrolled in November 2025. Pre-defined progression criteria will guide the decision to proceed to a full RCT: (1) successful recruitment of at least 70 % of eligible participants, (2) data completeness of outcome data ≥85 %, (3) fidelity of intervention delivery ≥80 %, and (4) absence of serious adverse events related to the intervention.

Recruitment will start in autumn of 2026 and last for 18 months, followed by six months of follow-up. The study process is expected to be completed by December 2028. Any protocol amendments will require Ethics Committee’s approval and will be documented on https://www.ClinicalTrials.gov.

## Discussion

The CARDIS trial is designed to improve the overall wellbeing and health of cardiac arrest survivors by providing a structured, accessible, and effective web-based support intervention. By using a digital format, the programme has the potential to reach a broad and diverse patient population, supporting cost-effectiveness and promoting high adherence. In contrast to existing clinician-led or schedule-based on-line interventions, CARDIS offers a flexible, self-guided format that can be accessed independently of time and place, enabling scalability and integration into diverse follow-up pathways.

This RCT builds on previous feasibility work and aims to evaluate the clinical impact of the intervention on recovery and reintegration into everyday life. A comprehensive process evaluation will provide valuable insights into adherence, social selection, and engagement, which are essential for future real-world implementation.

Findings from CARDIS may support the implementation of more accessible and standardised follow-up pathways by complementing existing care structures, thereby enhancing equity in long-term outcomes and HRQoL without requiring additional healthcare resources. Moreover, CARDIS adds a unique contribution to the growing survivorship literature by evaluating a co-created intervention in a multicentre RCT, generating novel evidence on how digital support can enhance life after cardiac arrest.

## CRediT authorship contribution statement

**Annette Waldemar:** Writing – review & editing, Project administration, Methodology, Investigation. **Johan Israelsson:** Writing – review & editing, Methodology, Investigation. **Katarina Heimburg:** Writing – review & editing, Methodology. **Erik Blennow Nordström:** Writing – review & editing, Methodology. **Per Nordberg:** Writing – review & editing, Methodology. **Anders Bremer:** Writing – review & editing, Methodology, Investigation. **Kristofer Årestedt:** Writing – review & editing, Supervision, Methodology, Investigation, Formal analysis. **Ingela Thylén:** Writing – original draft, Supervision, Project administration, Methodology, Investigation, Funding acquisition, Formal analysis, Conceptualization.

## Declaration of competing interest

The authors declare that they have no known competing financial interests or personal relationships that could have appeared to influence the work reported in this paper.
